# Causal Effects and Single‐Cell Microenvironmental Implications of Germline Variant‐Regulated Lactylation‐Related Pro‐Oncogenic Genes in Colorectal Cancer

**DOI:** 10.1155/humu/3085047

**Published:** 2026-06-17

**Authors:** Biao Sheng, Jianwei Wang

**Affiliations:** ^1^ Department of Surgery, The Fourth Affiliated Hospital of School of Medicine, International School of Medicine, International Institutes of Medicine, Zhejiang University, Yiwu, China, zju.edu.cn; ^2^ Department of Colorectal Surgery and Oncology (Key Laboratory of Cancer Prevention and Intervention, China National Ministry of Education), The Second Affiliated Hospital, Zhejiang University School of Medicine, Hangzhou, Zhejiang, China, zju.edu.cn; ^3^ Anhui Hospital of the Second Affiliated Hospital Zhejiang University School of Medicine (ZJUSAH-AH), China

**Keywords:** AXIN1, colorectal cancer, germline variant, GWAS analysis, lactylation, single-cell analysis

## Abstract

**Background:**

Colorectal cancer (CRC) is shaped by inherited genetic susceptibility, metabolic reprogramming, epigenetic regulation, and tumor microenvironment (TME) heterogeneity. Lactylation has recently emerged as an epigenetic mechanism that links lactate accumulation to chromatin remodeling and transcriptional regulation. However, the roles of lactylation‐related genes in CRC initiation and progression, particularly from the perspective of SNP‐based germline genetic variation, remain to be elucidated.

**Methods:**

We integrated GWAS analysis and single‐cell RNA sequencing to identify lactylation‐related pro‐oncogenic genes with potential genetic causal relevance and to further characterize their cellular localization and microenvironmental implications in CRC. GWAS uses inherited germline genetic variants, particularly SNPs associated with gene expression, as instrumental variables, thereby reducing confounding and reverse causation compared with conventional tumor expression‐based analyses.

**Results:**

GWAS analysis has identified germline variants in AXIN1, FASN, MLH1, and RAD50 that genetically predicted increased CRC risk. Single‐cell analysis was subsequently performed to evaluate the cell‐type‐specific distribution and functional relevance of these GWAS‐identified genes within CRC tissues. Among them, AXIN1 exhibited clearer differential expression and cell‐type‐specific distribution in CRC‐associated cell populations. AXIN1‐high cell populations were associated with metabolic adaptation, proliferative activity, lactylation‐related transcriptional programs, inflammatory responses, immune regulation, extracellular matrix remodeling, and TME adaptation. Besides, FASN was also confirmed as a genetically supported risk gene implicated in CRC metabolic reprogramming. MLH1 and RAD50 were retained as candidate risk genes at the germline causal level.

**Conclusion:**

This study integrates germline variant‐based causal inference with single‐cell microenvironmental interpretation, highlighting germline variant‐regulated lactylation in promoting colorectal cancer risk.

## 1. Introduction

Colorectal cancer (CRC) is one of the most common malignancies of the digestive system worldwide, and its incidence and mortality have remained high [1, 2]. Although advances in endoscopic screening, surgery, radiotherapy, chemotherapy, targeted therapy, and immunotherapy have substantially improved clinical outcomes in some patients, individuals with advanced CRC still face recurrence, metastasis, therapeutic resistance, and limited long‐term survival [3]. CRC is not driven by a single genetic event or signaling pathway. Rather, it results from the combined effects of genetic susceptibility, metabolic reprogramming, epigenetic regulation, immune escape, and dynamic remodeling of the TME [4, 5]. Therefore, identifying key molecules with potential causal relevance that also reflect tumor‐cell states and microenvironmental remodeling is essential for clarifying the mechanisms of CRC progression and discovering new therapeutic targets.

Metabolic reprogramming is one of the major biological hallmarks of CRC. Tumor cells frequently exhibit enhanced glycolytic activity and accumulate large amounts of lactate within the local microenvironment [6, 7]. Zhang et al. demonstrated that lactate can serve as a precursor for histone lysine lactylation and directly stimulate gene transcription on chromatin, thereby converting metabolic‐state changes into sustained transcriptional regulatory signals [8]. This discovery provides a new theoretical basis for understanding how tumor metabolic abnormalities influence epigenetic states and cellular functions. In the tumor context, lactylation may represent an important mechanism linking enhanced glycolysis, epigenetic remodeling, and tumor microenvironmental adaptation [9]. Existing studies have shown that both histone and nonhistone lactylation can participate in tumor‐cell proliferation, invasion, immune escape, angiogenesis, and therapeutic resistance [10]. In recent years, increasing attention has been paid to lactate metabolism and lactylation regulation in CRC, suggesting that lactylation may be involved in cancer‐cell stemness maintenance, cell‐death evasion, chemoresistance, and microenvironmental remodeling [11, 12]. Despite these advances, an important knowledge gap remains. Most current studies on lactylation‐related genes in CRC have mainly been based on tumor tissue expression analysis, prognostic model construction, correlation with clinical features, or functional validation of individual molecules [13, 14].

GWAS provides a suitable methodological strategy for addressing this issue. GWAS uses inherited germline genetic variants as instrumental variables to estimate the potential causal effect of an exposure on a disease outcome [15, 16]. In transcriptome‐wide GWAS, expression quantitative trait loci (eQTLs), most commonly SNPs associated with gene expression, are used to represent genetically predicted gene expression [17]. Nevertheless, GWAS alone cannot determine which cell populations express these candidate genes in tumor tissues or how they may participate in the CRC microenvironment [18]. Bulk transcriptomic analyses may obscure whether a risk‐associated gene is mainly expressed in tumor epithelial cells, immune cells, stromal compartments, or specific microenvironmental states [19]. In this study, we integrate GWAS analysis and single‐cell technology to determine the cellular distribution, functional state, and potential microenvironmental role in CRC, providing a new framework to germline variant‐regulated lactylation in promoting CRC risk.

## 2. Materials and Methods

### 2.1. Study Design

This study is aimed at integrating two‐sample GWAS analysis with GWAS data to systematically investigate the potential causal effects and cell‐type‐specific roles of lactylation‐related pro‐oncogenic genes in CRC. The overall workflow included construction of a lactylation‐related gene set, selection of eQTL instrumental variables, two‐sample GWAS analysis, identification of GWAS‐positive pro‐oncogenic candidate genes, processing of GWAS data, and downstream single‐cell analyses of gene expression localization, functional states, and cell–cell communication patterns. The study design followed the three core assumptions of GWAS: the genetic instrumental variables should be strongly associated with the exposure; the instrumental variables should not be associated with potential confounders; and the instrumental variables should influence the outcome only through the exposure. In the GWAS stage, genes whose genetically predicted expression was associated with increased CRC risk were identified. In the single‐cell analysis stage, these candidate genes were further examined to determine their cellular sources, expression distribution, and potential functional states within the CRC tumor microenvironment (TME).

### 2.2. Data Sources

The CRC GWAS outcome data used in this study were obtained from the IEU OpenGWAS database, with the dataset ID ebi‐a‐GCST90018808 and the URL https://opengwas.io/datasets/ebi-a-GCST90018808. This dataset corresponds to the phenotype “CRC,” uses the HG19/GRCh37 genome build, and is based on a European population. The eQTL data of lactylation‐related genes were used as exposure data to evaluate potential causal relationships between genetically predicted gene expression and CRC risk.

GWAS data were obtained from the GEO database under accession number GSE132465, with the URL https://www.ncbi.nlm.nih.gov/geo/query/acc.cgi?acc=GSE132465. This dataset is titled “Single cell 3 ^′^ RNA sequencing of 23 Korean CRC patients” and includes 23 primary CRC tissue samples and 10 matched normal mucosa samples from 23 Korean CRC patients. It provides a single‐cell transcriptomic atlas of CRC and normal mucosal tissues, enabling analysis of intrinsic tumor‐cell characteristics, TME composition, and potential intercellular interactions. No TCGA, GEO bulk RNA‐seq, or other bulk transcriptomic datasets were used in this study.

### 2.3. Construction of the Lactylation‐Related Gene Set

Lactylation‐related genes were collected by reviewing published studies on lactate metabolism, lysine lactylation, histone lactylation, nonhistone lactylation, and tumor‐associated lactylation. A total of 161 lactylation‐related genes were collected [20, 21]. The included genes were mainly involved in lactate production, lactate transport, lactate‐mediated signaling, regulation of lactylation modification, tumor metabolic reprogramming, and lactylation‐related biological functions. All gene names were standardized to official gene symbols, and duplicated genes, nonstandard gene symbols, and genes that could not be matched to available eQTL data were removed. The curated lactylation‐related gene list was then intersected with genes with available eQTL instrumental variables, and only genes eligible for GWAS analysis were retained. Based on subsequent GWAS screening, AXIN1, FASN, MLH1, and RAD50 were identified as the key lactylation‐related pro‐oncogenic candidate genes for further analysis.

### 2.4. Instrumental Variable Selection and Data Harmonization

In the two‐sample GWAS analysis, eQTL variants of lactylation‐related genes were used as instrumental variables. Instrumental variable selection followed the principles of relevance, independence, and exclusion restriction. First, SNPs significantly associated with target gene expression were selected as candidate instrumental variables, with a significance threshold of *p* < 5 × 10^−8^. Linkage disequilibrium clumping was then performed to remove highly correlated SNPs, with the parameters set at *r* ^2^ < 0.001 and a clumping window of 10,000 kb. Finally, the F statistic was calculated to evaluate the strength of each instrumental variable. SNPs with an F statistic less than 10 were considered potentially affected by weak instrumental variable bias and were excluded from subsequent analyses.

During harmonization of exposure and outcome data, the effect alleles of SNPs were aligned to ensure consistent effect directions between exposure and outcome datasets. Palindromic SNPs with ambiguous allele orientation were removed when allele frequency information was insufficient to determine the correct direction. The harmonized SNPs were then used for GWAS effect estimation, sensitivity analyses, and visualization.

### 2.5. Two‐Sample GWAS Analysis

Two‐sample GWAS analysis was performed using the TwoSampleMR package in R. Genetically predicted expression levels of lactylation‐related genes were used as exposures, and CRC was used as the outcome. GWAS analyses were conducted using the inverse‐variance weighted method, Mendelian randomization (MR)‐Egger regression, weighted median method, simple mode method, and weighted mode method. The IVW method was used as the primary approach for causal effect estimation, whereas the other methods were used to evaluate the consistency and robustness of the direction of effect. The TwoSampleMR framework supports GWAS estimation as well as leave‐one‐out analysis to assess whether the observed association is driven by a single SNP [22, 23]. If the IVW analysis showed an OR > 1 and *p* < 0.05, the genetically predicted increase in gene expression was considered to be associated with increased CRC risk, and the gene was defined as a lactylation‐related pro‐oncogenic candidate gene.

Based on this screening criterion, AXIN1, FASN, MLH1, and RAD50 were included in subsequent single‐cell analyses. For AXIN1, 21 SNPs were included as instrumental variables, and IVW analysis showed that genetically predicted higher AXIN1 expression was significantly associated with increased CRC risk, with an OR of 1.096, 95% CI of 1.039–1.157, and *p* = 0.000812. For FASN, 22 SNPs were included, and the IVW analysis showed an OR of 1.072, 95% CI of 1.016–1.130, and *p* = 0.0102. For MLH1, 16 SNPs were included, and the IVW analysis showed an OR of 1.056, 95% CI of 1.007–1.107, and *p* = 0.0253. For RAD50, 8 SNPs were included, and the IVW analysis showed an OR of 1.124, 95% CI of 1.003–1.259, and *p* = 0.0437.

### 2.6. GWAS Sensitivity Analyses

To evaluate the reliability and robustness of the GWAS results, heterogeneity testing, horizontal pleiotropy assessment, and leave‐one‐out analysis were performed. Heterogeneity was assessed using Cochran′s Q statistic, with *p* < 0.05 indicating potential heterogeneity among instrumental variables. Horizontal pleiotropy was evaluated using the MR‐Egger intercept test. *p* < 0.05 for the intercept suggested that the GWAS results might be affected by directional horizontal pleiotropy. Leave‐one‐out analysis was performed by sequentially removing each SNP and recalculating the GWAS effect, thereby assessing whether the overall causal estimate was driven by a single instrumental variable. If the direction of effect remained consistent after removal of any individual SNP, the GWAS result was considered robust. Scatter plots, forest plots, funnel plots, and leave‐one‐out plots were generated to visualize the GWAS results.

### 2.7. Processing of GWAS Data

The GSE132465 GWAS data were processed using the Seurat package in R. The raw UMI count matrix and cell annotation files were first imported to construct a Seurat object. Quality control was performed based on the number of detected genes, UMI counts, and the percentage of mitochondrial genes in each cell. Low‐quality cells, putative empty droplets, and potential doublets were removed according to quality‐control thresholds. Since preliminary quality control had already been performed in the original GSE132465 study, necessary secondary quality control was conducted based on the actual expression matrix used in this study. The expression matrix was then normalized using the NormalizeData function, highly variable genes were identified using FindVariableFeatures, and data scaling was performed using ScaleData. Principal component analysis was conducted using RunPCA, and the appropriate number of principal components was selected based on the proportion of variance explained or the ElbowPlot results. Cell neighborhood graph construction and clustering were performed using FindNeighbors and FindClusters, respectively, followed by two‐dimensional visualization using RunUMAP.

### 2.8. Integrated Downstream Single‐Cell Analysis

After quality control, normalization, dimensionality reduction, and clustering, major cell types were confirmed using the cell annotation information provided by GSE132465 together with canonical cell‐type marker genes. The major cell populations were annotated according to canonical lineage markers. Epithelial cells were identified by EPCAM, KRT8, and KRT18; T cells by CD3D, CD3E, and TRAC; B cells by MS4A1, CD79A, and CD79B; myeloid cells by LYZ, CD68, and CD14; stromal cells by COL1A1, DCN, and LUM; and mast cells by TPSAB1, TPSB2, and CPA3. These marker genes were used to support the reliability of cell‐type annotation and to facilitate the interpretation of cell‐type‐specific expression patterns in subsequent analyses.

The expression levels of AXIN1, FASN, MLH1, and RAD50 were then extracted across different cell types, and their cellular distribution patterns in CRC and normal mucosal tissues were visualized using UMAP plots, FeaturePlot, DotPlot, and violin plots. To further evaluate lactylation‐related transcriptional states, a single‐cell lactylation score was constructed based on the lactylation‐related gene set, and differences in this score were compared across tissue sources and cell populations. Differential expression analysis was subsequently performed according to candidate gene expression levels or lactylation score stratification, and GO and KEGG enrichment analyses were conducted using the resulting differentially expressed genes to explore potential biological processes. Finally, CellChat was used to analyze ligand–receptor interactions among different cell populations, with a particular focus on potential communication patterns between lactylation‐related pro‐oncogenic gene‐high cell populations and immune, stromal, or epithelial cells. CellChat is designed to quantitatively infer and analyze intercellular communication networks from single‐cell RNA‐seq data. This analysis was used to explore the potential intercellular interaction mechanisms through which AXIN1, FASN, MLH1, and RAD50 may contribute to CRC microenvironment remodeling.

### 2.9. Statistical Analysis

All statistical analyses were performed in R. Two‐sample GWAS analyses were conducted using the TwoSampleMR package. GWAS data were processed using the Seurat package. Functional enrichment analyses were performed using the clusterProfiler, enrichplot, and https://org.hs.eg/.db packages. Cell–cell communication analysis was performed using the CellChat package. For continuous variables, comparisons between two groups were performed using the Wilcoxon rank‐sum test or Student′s t test, whereas comparisons among multiple groups were performed using the Kruskal–Wallis test or one‐way analysis of variance. Correlation analysis was conducted using Spearman correlation analysis. Unless otherwise specified, all statistical tests were two‐sided, and *p* < 0.05 was considered statistically significant. For multiple comparisons, the Benjamini–Hochberg method was used to control the false discovery rate, and adjusted *p* < 0.05 was considered statistically significant.

Single‐cell RNA‐seq analysis was performed using R software Version 4.3.1. The main R packages used in this study included Seurat (v5.0.1) for quality control, normalization, dimensionality reduction, clustering, cell‐type annotation, and visualization; Matrix (v1.6‐5) for sparse matrix processing; dplyr (v1.1.4) and data. Table (v1.15.4) for data manipulation; ggplot2 (v3.5.1), patchwork (v1.2.0), and pheatmap (v1.0.12) for data visualization; clusterProfiler (v4.10.1), https://org.hs.eg/.db (v3.18.0), and enrichplot (v1.22.0) for functional enrichment analysis; and CellChat (v2.1.2) for cell–cell communication analysis. If pathway‐level activity analysis was performed, GSVA (v1.50.5), msigdbr (v7.5.1), and limma (v3.58.1) were used for gene set‐based pathway scoring and downstream statistical analysis.

## 3. Results

### 3.1. GWAS Analysis Identified Lactylation‐Related Candidate Genes Associated With Increased CRC Risk

**Figure 1:**
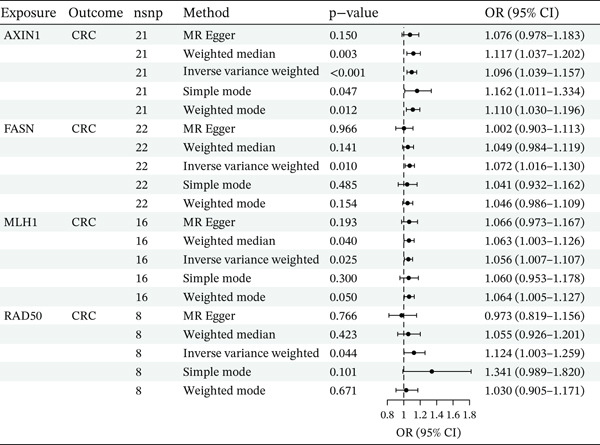
GWAS analysis of lactylation‐related candidate genes associated with CRC risk.

To identify lactylation‐related pro‐oncogenic candidate genes potentially involved in CRC development from the perspective of genetic causality, this study used eQTLs of lactylation‐related genes as exposures and CRC as the outcome. The GWAS results showed that genetically predicted higher expression of AXIN1, FASN, MLH1, and RAD50 was associated with increased CRC risk, suggesting that these genes may represent lactylation‐related CRC risk candidates (Figure 1). Among them, AXIN1 exhibited the most stable and consistent risk effect. Based on 21 SNP instrumental variables, IVW analysis showed that genetically predicted higher AXIN1 expression was significantly associated with increased CRC risk, with an OR of 1.096, 95% CI of 1.039–1.157, and *p* < 0.001. Weighted median analysis also showed a significant positive association, with an OR of 1.117, 95% CI of 1.037–1.202, and *p* = 0.003. Simple mode and weighted mode analyses further supported an increased CRC risk associated with AXIN1, with ORs of 1.162 and 1.110 and *p* values of 0.047 and 0.012, respectively. Although the GWAS Egger analysis did not reach statistical significance, the effect direction remained positive, indicating good consistency of the AXIN1 GWAS results across different methods (Figure 1).

FASN was also identified as a candidate gene associated with CRC risk. Based on 22 SNP instrumental variables, IVW analysis showed that genetically predicted higher FASN expression was significantly associated with increased CRC risk, with an OR of 1.072, 95% CI of 1.016–1.130, and *p* = 0.010. Although GWAS Egger, weighted median, simple mode, and weighted mode analyses did not reach statistical significance, their effect estimates were generally in the positive direction, suggesting that FASN may be supported mainly by the IVW model as a lactylation‐related CRC risk gene. For MLH1, 16 SNPs were used as instrumental variables. The IVW result showed that genetically predicted higher MLH1 expression was associated with increased CRC risk, with an OR of 1.056, 95% CI of 1.007–1.107, and *p* = 0.025. Weighted median analysis also reached statistical significance, with an OR of 1.063, 95% CI of 1.003–1.126, and *p* = 0.040. For RAD50, 8 SNPs were included as instrumental variables. IVW analysis showed that genetically predicted higher RAD50 expression was associated with increased CRC risk, with an OR of 1.124, 95% CI of 1.003–1.259, and *p* = 0.044. However, the other GWAS methods did not reach statistical significance, suggesting that although RAD50 could be retained as a GWAS‐positive candidate gene, the consistency of its causal evidence was weaker than that of AXIN1 (Figure 1).

Forest plot showing the causal estimates of genetically predicted expression of AXIN1, FASN, MLH1, and RAD50 on CRC risk using five GWAS methods, including MR‐Egger, weighted median, inverse variance weighted, simple mode, and weighted mode. The number of SNPs used for each exposure, *p* values, and odds ratios with 95% confidence intervals are shown. The inverse variance weighted method was used as the primary analytical approach. AXIN1, FASN, MLH1, and RAD50 showed positive causal estimates in the IVW analysis, suggesting that genetically predicted higher expression of these lactylation‐related genes was associated with increased CRC risk. AXIN1 exhibited the most consistent positive association across multiple GWAS methods.

**Figure 2:**
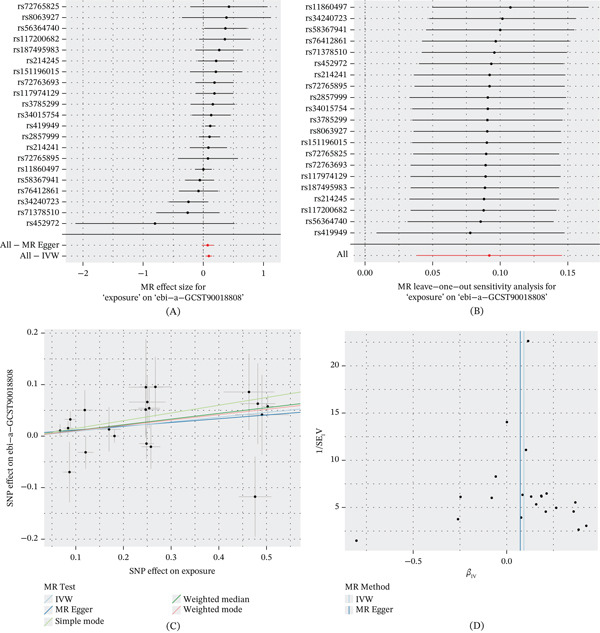
Sensitivity analyses and visualization of the GWAS results for AXIN1. Sensitivity analyses were performed to evaluate the robustness of the causal association between genetically predicted AXIN1 expression and CRC risk. (A) Single‐SNP forest plot showing the causal effect estimates of individual SNPs and the combined estimates from MR‐Egger and IVW analyses. (B) Leave‐one‐out analysis showing that sequential removal of each SNP did not substantially alter the overall positive causal estimate, indicating that the AXIN1–CRC association was not driven by a single influential variant. (C) Scatter plot showing the relationship between SNP effects on AXIN1 expression and SNP effects on CRC risk across different GWAS methods, with fitted lines indicating the estimated causal directions. (D) Funnel plot displaying the distribution of SNP‐specific causal estimates, with no obvious asymmetry by visual inspection, suggesting no strong evidence of directional bias.

Given that AXIN1 showed a relatively consistent positive effect across multiple GWAS methods, additional sensitivity analyses and visualization were performed for AXIN1. The single‐SNP forest plot showed that most SNP‐specific effect estimates were distributed in the positive risk direction, and the overall IVW estimate remained stable, suggesting that the positive association between AXIN1 and CRC risk was not primarily offset or driven by a few variants with opposite effects (Figure 2A). Leave‐one‐out analysis showed that the overall GWAS effect between AXIN1 and CRC risk remained positive after sequential removal of each SNP, indicating that the result was not obviously dominated by a single influential SNP (Figure 2B). The scatter plot further showed that the fitted slopes of different GWAS methods were generally positive, supporting a positive relationship between genetically predicted AXIN1 expression and increased CRC risk (Figure 2C). In the funnel plot, the SNP effect estimates did not show an obvious asymmetric distribution, suggesting no clear visual evidence of directional bias in the AXIN1 GWAS results (Figure 2D).

Overall, GWAS analysis identified AXIN1, FASN, MLH1, and RAD50 as lactylation‐related candidate genes associated with increased CRC risk from a germline variant‐based causal inference perspective. Because GWAS uses inherited SNPs as instrumental variables, these findings suggest that the risk associations observed here may reflect SNP‐based germline regulatory alterations rather than simple tumor‐associated transcriptional changes. Among the four candidates, AXIN1 showed the strongest statistical association in the IVW analysis and maintained a consistent risk direction across weighted median, simple mode, and weighted mode analyses. In addition, leave‐one‐out and funnel plot analyses further supported the robustness of the AXIN1 result. Therefore, AXIN1 was prioritized as the key germline variant‐supported candidate gene for subsequent single‐cell transcriptomic and mechanistic analyses.

### 3.2. Single‐Cell Transcriptomic Analysis Reveals the Cellular Distribution of GWAS‐Positive Genes in the CRC Microenvironment

**Figure 3:**
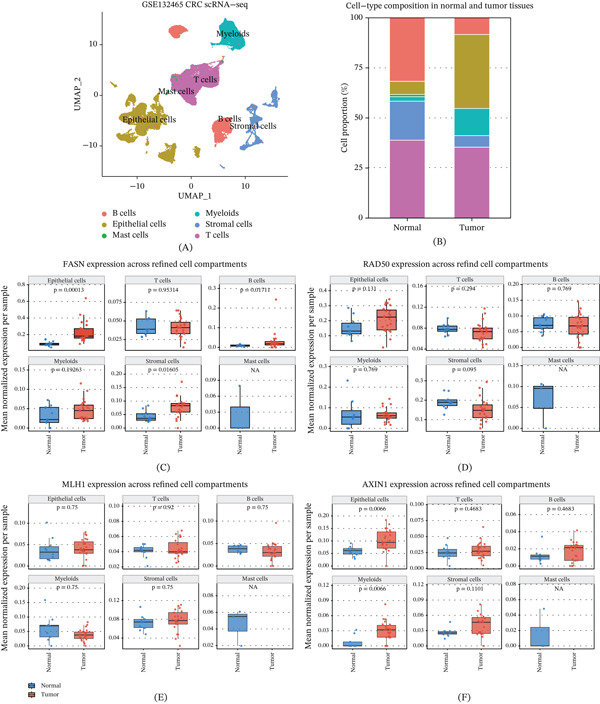
Single‐cell transcriptomic characterization of GWAS‐positive lactylation‐related candidate genes in CRC tissues. (A) UMAP visualization of the GSE132465 CRC GWAS dataset, showing the major annotated cell populations, including epithelial cells, T cells, B cells, myeloid cells, stromal cells, mast cells, and other immune compartments. (B) Stacked bar plot showing the cell‐type composition of normal mucosal and tumor tissues, indicating altered cellular proportions in the CRC microenvironment. (C–F) Boxplots showing the normalized expression levels of GWAS‐positive lactylation‐related candidate genes across refined cell compartments in normal and tumor tissues. (C) FASN expression was significantly increased in tumor epithelial cells and also showed tumor‐associated elevation in B cells and stromal cells. (D) RAD50 showed no obvious cell‐type‐specific differential expression between normal and tumor tissues. (E) MLH1 displayed a relatively stable expression pattern across major cell compartments. (F) AXIN1 was significantly upregulated in tumor epithelial cells and myeloid cells, suggesting a tumor‐associated and cell‐type‐specific expression pattern. These results indicate that AXIN1 exhibits clearer single‐cell‐level differential expression than MLH1 and RAD50 and was therefore prioritized for subsequent mechanistic analyses.

To further characterize the cellular sources and expression heterogeneity of the lactylation‐related candidate genes identified by GWAS analysis in CRC tissues, we performed downstream analyses using the GSE132465 GWAS dataset. UMAP‐based dimensionality reduction was used to visualize the single‐cell population structure. The cells in GSE132465 were classified into major cell types, including epithelial cells, T cells, B cells, myeloid cells, stromal cells, and mast cells. These cell populations showed relatively clear separation in the low‐dimensional space, indicating that this dataset effectively captured the cellular heterogeneity of CRC tissues (Figure 3A). Further comparison of cellular composition between normal mucosal and tumor tissues revealed marked alterations in cell‐type proportions. Compared with normal tissues, tumor tissues exhibited a substantially increased proportion of epithelial cells, accompanied by corresponding changes in immune and stromal cell populations. These findings suggest prominent tumor‐cell expansion and microenvironmental remodeling in CRC tissues (Figure 3B).

Next, we evaluated the expression differences of the GWAS‐positive genes AXIN1, FASN, MLH1, and RAD50 across different cell types and between normal and tumor tissues. FASN showed a significant tumor‐associated increase in epithelial cells, with markedly higher expression in tumor epithelial cells than in normal mucosal epithelial cells (*p* = 0.00013; Figure 3C). In addition, FASN also showed increased expression in B cells and stromal cells from tumor tissues, with *p* values of 0.01711 and 0.01605, respectively, whereas no significant difference was observed in T cells or myeloid cells. These results indicate that FASN expression changes were mainly concentrated in tumor epithelial cells and selected microenvironmental compartments, consistent with its known roles in lipid metabolism and tumor progression.

In contrast, RAD50 did not show obvious expression differences across cell types. Although RAD50 exhibited a modest increasing trend in tumor epithelial cells, the difference did not reach statistical significance in epithelial cells (*p* = 0.131), nor were significant differences observed in T cells, B cells, myeloid cells, or stromal cells (Figure 3D). Similarly, MLH1 displayed a relatively stable expression pattern, with no evident differences between normal and tumor tissues in epithelial cells, T cells, B cells, myeloid cells, or stromal cells (Figure 3E). These results suggest that although RAD50 and MLH1 were associated with increased CRC risk in the GWAS analysis, they did not exhibit clear cell‐type‐specific differential expression patterns in the GSE132465 single‐cell data. Therefore, these genes may be more appropriately considered candidate risk genes at the level of genetic causal inference rather than primary targets for single‐cell mechanistic analysis in this study.

Notably, AXIN1 showed relatively clear tumor‐associated expression characteristics at the single‐cell level. AXIN1 expression was significantly higher in tumor epithelial cells than in normal mucosal epithelial cells (*p* = 0.0066), suggesting that AXIN1 may be associated with intrinsic state changes in CRC tumor cells (Figure 3F). In addition, AXIN1 was also significantly increased in myeloid cells (p = 0.006), indicating that AXIN1‐related signals may not be restricted to tumor epithelial cells but may also be linked to altered immune‐cell states within the TME. Although AXIN1 showed an increasing trend in stromal cells, this difference did not reach statistical significance (*p* = 0.1101), and no significant differences were observed in T cells or B cells (Figure 3F). These findings indicate that AXIN1 exhibits a relatively distinct cell‐type‐specific expression pattern in CRC tissues, particularly in epithelial and myeloid cell compartments.

Taken together, single‐cell transcriptomic analysis revealed that the four lactylation‐related genes identified by GWAS displayed heterogeneous expression patterns within CRC tissues. FASN was significantly upregulated in tumor epithelial cells, which was consistent with its previously reported role in CRC metabolic reprogramming and tumor progression. MLH1 and RAD50 were retained as germline variant‐supported risk genes at the GWAS level, but they did not show obvious cell‐type‐specific differential expression in the single‐cell data. In contrast, AXIN1 not only exhibited the most stable germline SNP‐supported risk effect in GWAS analysis but also showed clearer tumor‐associated and cell‐type‐specific expression characteristics. Therefore, AXIN1 was further selected as the key candidate for downstream analyses of lactylation‐related transcriptional scores, functional enrichment, and cell–cell communication mechanisms.

### 3.3. AXIN1 Is Enriched in Tumor‐Derived SPP1^+^ Myeloid Cells and Is Associated With Inflammatory Responses and Cell–Cell Communication Remodeling

**Figure 4:**
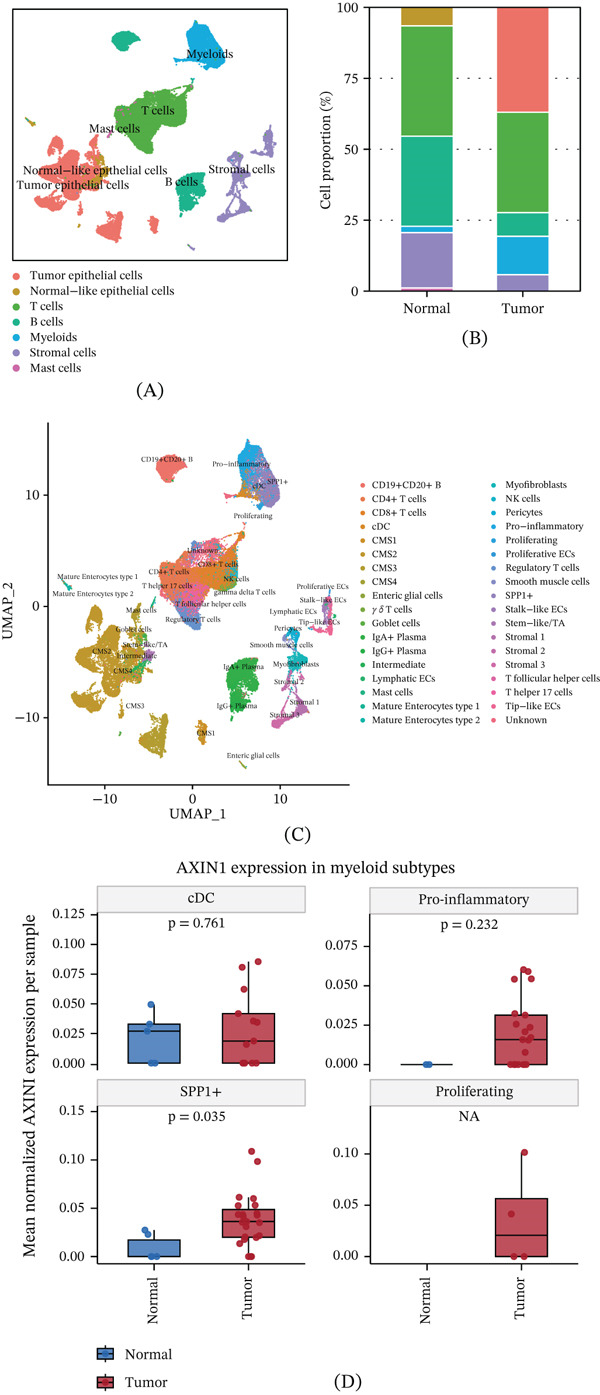
Refined single‐cell annotation and AXIN1 expression in myeloid subclusters. (A) UMAP visualization of refined cell‐type annotation in the CRC single‐cell data. (B) Cell‐type composition of normal mucosal and tumor tissues. (C) UMAP visualization of refined cell subpopulations, including epithelial, immune, stromal, and myeloid subsets. (D) AXIN1 expression across myeloid subclusters in normal and tumor tissues. AXIN1 was significantly increased in tumor‐derived SPP1^+^ myeloid cells, suggesting a preferential association between AXIN1 expression and the SPP1^+^ myeloid cell state in CRC.

To further define the specific cellular niche of AXIN1 within the CRC TME, we performed refined clustering of the single‐cell data based on the major cell‐type annotations. UMAP analysis showed that the cells could be further classified into tumor epithelial cells, normal‐like epithelial cells, T cells, B cells, myeloid cells, stromal cells, and mast cells. Compared with normal mucosal tissues, tumor tissues exhibited a higher proportion of tumor epithelial cells, accompanied by marked alterations in immune and stromal cell composition, suggesting substantial cellular remodeling and microenvironmental heterogeneity in CRC tissues (Figure 4A,B).

With further refined annotation, myeloid cells in CRC tissues were subdivided into cDC, proinflammatory, SPP1^+^, and proliferating subclusters. Since the previous analysis showed that AXIN1 was significantly increased in myeloid cells from tumor tissues, we further compared AXIN1 expression across different myeloid subclusters. AXIN1 was significantly upregulated in tumor‐derived SPP1^+^ myeloid cells, with higher expression in the tumor group than in the normal group (*p* = 0.035; Figure 4C). In contrast, AXIN1 expression did not differ significantly between tumor and normal tissues in cDC and proinflammatory myeloid cells, with *p* values of 0.761 and 0.232, respectively. Proliferating myeloid cells were mainly derived from tumor tissues, and there was no sufficient normal counterpart for stable comparison (Figure 4C). These findings suggest that the tumor‐associated increase in AXIN1 within myeloid cells is not uniformly distributed across all myeloid subclusters but is preferentially concentrated in the SPP1^+^ myeloid cell state.

**Figure 5:**
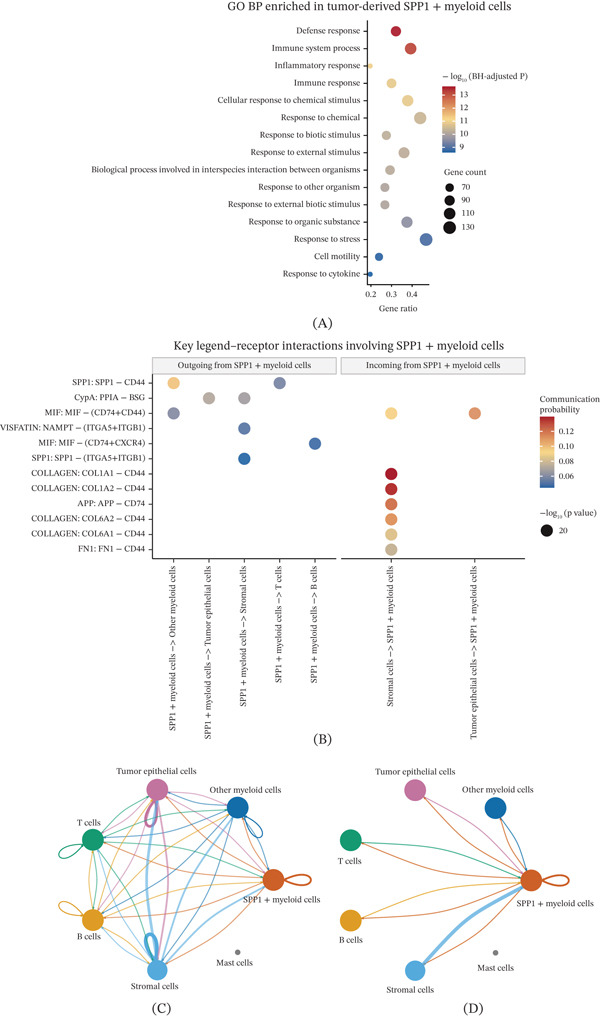
Functional enrichment and cell–cell communication features of SPP1^+^ myeloid cells. (A) GO biological process enrichment analysis of tumor‐derived SPP1^+^ myeloid cells, showing enrichment of immune response, inflammatory response, stress response, and cell motility‐related processes. (B) Key ligand–receptor interactions involving SPP1^+^ myeloid cells as signal senders or receivers. (C) Global cell–cell communication network among major CRC microenvironmental cell types. (D) Communication network centered on SPP1^+^ myeloid cells. These results indicate that SPP1^+^ myeloid cells may contribute to CRC microenvironment remodeling through inflammatory, immune‐related, and stromal interaction networks.

To further characterize the potential functional features of tumor‐derived SPP1^+^ myeloid cells, GO biological process enrichment analysis was performed using their associated differentially expressed genes. The results showed that tumor‐derived SPP1^+^ myeloid cells were significantly enriched in biological processes including defense response, immune system process, inflammatory response, immune response, cellular response to chemical stimulus, response to stress, response to cytokine, and cell motility (Figure 5A). These enriched processes indicate that SPP1^+^ myeloid cells are not merely a myeloid subpopulation but represent an active TME al cell state characterized by inflammatory responses, immune regulation, stress adaptation, and cell migration. Together with the increased AXIN1 expression in tumor‐derived SPP1^+^ myeloid cells, these findings suggest that AXIN1 may be associated with a proinflammatory myeloid state and microenvironmental adaptation in CRC.

Further cell–cell communication analysis revealed that SPP1^+^ myeloid cells exhibited strong intercellular interaction potential within the CRC microenvironment. The ligand–receptor interaction bubble plot showed that, as signal senders, SPP1^+^ myeloid cells could communicate with tumor epithelial cells, stromal cells, T cells, B cells, and other myeloid cells through several signaling axes, including SPP1–CD44, SPP1–ITGA5/ITGB1, MIF–CD74/CD44, MIF–CD74/CXCR4, NAMPT–ITGA5/ITGB1, PPIA–BSG, and multiple COLLAGEN–CD44 interactions (Figure 5B). These signaling pathways are closely related to cell adhesion, inflammatory responses, stromal remodeling, immune regulation, and tumor‐cell migration, suggesting that SPP1^+^ myeloid cells may contribute to CRC microenvironmental remodeling through multiple ligand–receptor axes.

Meanwhile, SPP1^+^ myeloid cells could also act as signal receivers and form bidirectional communication networks with tumor epithelial and stromal cells. Incoming signal analysis showed that stromal cells and tumor epithelial cells could act on SPP1^+^ myeloid cells through COLLAGEN–CD44, APP–CD74, FN1–CD44, and MIF‐related signaling axes, suggesting that SPP1^+^ myeloid cells may be jointly regulated by tumor cells and the stromal microenvironment (Figure 5B). The global cell–cell communication network further showed extensive interactions between SPP1^+^ myeloid cells and tumor epithelial cells, stromal cells, other myeloid cells, T cells, and B cells, with particularly prominent communication with stromal and tumor epithelial cells (Figure 5C,D).

In summary, refined single‐cell analysis showed that AXIN1 was significantly increased in tumor‐derived SPP1^+^ myeloid cells. This cell population was characterized by inflammatory responses, immune regulation, stress adaptation, cell migration, and active interaction with epithelial and stromal compartments. Cell–cell communication analysis further suggested that AXIN1‐high SPP1^+^ myeloid cells may participate in CRC microenvironmental remodeling through ligand–receptor axes involving SPP1, MIF, COLLAGEN, APP, and FN1. These findings link germline variant‐supported lactylation‐related risk genes to specific cellular states within the CRC TME. In particular, AXIN1 may mark or participate in the inflammatory and stromal‐interaction state of SPP1^+^ myeloid cells, providing a focused direction for future mechanistic validation.

## 4. Discussion

In this study, we present an integrative framework that bridges germline genetic susceptibility, epigenetic metabolism, and single‐cell TME architecture to interrogate lactylation‐related pro‐oncogenic mechanisms in CRC. By leveraging two‐sample MR with eQTLs as instrumental variables, followed by high‐resolution single‐cell transcriptomic mapping, we identified AXIN1, FASN, MLH1, and RAD50 as lactylation‐associated genes whose genetically predicted expression correlates with increased CRC risk. Among these, AXIN1 emerged as the most robust candidate, exhibiting consistent causal signals across multiple MR methods and preferential enrichment in tumor‐derived SPP1^+^ myeloid cells—a cellular state implicated in inflammatory remodeling, stromal crosstalk, and immune modulation within the CRC niche. These findings advance the conceptualization of lactylation from a correlative metabolic‐epigenetic phenomenon toward a germline‐informed, cell‐type‐resolved regulatory axis with potential causal relevance to CRC pathogenesis.

Conventional transcriptomic studies of lactylation in CRC have largely relied on differential expression analysis, prognostic modeling, or functional validation of individual molecules [24, 25]. Although informative, such approaches cannot readily distinguish whether observed transcriptional alterations represent upstream drivers of tumorigenesis or downstream consequences of metabolic reprogramming, inflammatory signaling, or cellular composition shifts. By contrast, our GWAS framework exploits the random allocation of germline SNPs at conception to approximate a natural randomized experiment: because these variants are fixed prior to disease onset and are largely independent of environmental confounders, they offer a powerful lens to evaluate whether genetically regulated expression of lactylation‐related genes may causally influence CRC susceptibility [26, 27]. The identification of AXIN1, FASN, MLH1, and RAD50 through this lens suggests that inherited regulatory variation in lactylation‐associated pathways may contribute to interindividual differences in CRC risk, independent of tumor‐stage–dependent transcriptional noise. MLH1 and RAD50 are involved in mismatch repair and DNA double‐strand break response, respectively, but they did not show obvious cell‐type–specific differential expression in the single‐cell data analyzed here [28–30]. Therefore, these genes were retained as germline variant‐supported candidate risk genes, whereas AXIN1 was prioritized for further microenvironmental interpretation because it showed clearer tumor‐associated and cell‐type‐related expression characteristics.

AXIN1 is classically characterized as a scaffold protein within the *β*‐catenin destruction complex, serving as a negative regulator of Wnt/*β*‐catenin signaling—a pathway frequently hyperactivated in CRC [31, 32]. Paradoxically, our GWAS analysis indicated that genetically predicted higher AXIN1 expression associates with elevated CRC risk. This apparent contradiction underscores the context‐dependent nature of AXIN1 biology. Emerging evidence suggests that AXIN1 can engage in Wnt‐independent functions, including modulation of p53 stability, TGF‐*β* signaling, and metabolic stress responses [33, 34]. In this study, the GWAS result suggested that genetically predicted higher AXIN1 expression was associated with increased CRC risk, whereas single‐cell analysis further showed that AXIN1 was enriched in specific CRC‐associated cellular states, particularly tumor‐derived SPP1^+^ myeloid cells. This observation should not be interpreted as a simple linear pro‐oncogenic effect of AXIN1. Rather, it may reflect a more complex relationship in which germline variant‐regulated AXIN1 expression interacts with tumor‐cell state, inflammatory signaling, lactylation‐related transcriptional programs, and microenvironmental remodeling [35, 36].

A distinctive strength of this study lies in its sequential integration of germline causal inference and single‐cell resolution. By first filtering lactylation‐related genes through a germline variant–based MR lens, we reduce the likelihood of prioritizing transcriptional changes that are merely epiphenomena of tumor burden or treatment exposure. Subsequent single‐cell mapping then contextualizes these genetically supported candidates within the cellular ecosystem of CRC, revealing potential effector compartments and intercellular communication networks. This two‐tiered strategy may serve as a generalizable template for interrogating other metabolism‐epigenetics‐immunity axes in cancer. From a translational perspective, AXIN1‐high SPP1^+^ myeloid cells could represent a therapeutically actionable niche: targeting lactylation writers/erasers, disrupting SPP1‐mediated stromal crosstalk, or reprogramming myeloid cell states may synergize with existing immunotherapies or metabolic inhibitors in CRC. Moreover, germline variant profiles of lactylation‐related genes could inform risk stratification or patient selection for metabolism‐targeted clinical trials.

Several limitations warrant consideration. First, the GWAS/eQTL data and single‐cell dataset were derived from distinct populations (European and Korean, respectively), introducing potential ancestry‐related heterogeneity in genetic architecture and cellular composition. Although GWAS is robust to population stratification when properly controlled, cross‐population validation will strengthen generalizability. Second, our analyses were conducted at the transcriptomic level; direct measurement of protein expression, lactylation modifications, and spatial colocalization of AXIN1 with SPP1^+^ myeloid cells in human CRC specimens remains an important next step. Third, although MR minimizes confounding and reverse causation, residual pleiotropy cannot be entirely excluded despite sensitivity analyses. Finally, functional validation—through CRISPR‐based perturbation, lactylation‐specific antibodies, or organoid coculture systems—will be essential to establish mechanistic causality. Future work should also explore whether lactylation‐related germline variants interact with environmental factors (diet, microbiome) to modulate CRC risk, and whether AXIN1 or its downstream effectors can be targeted pharmacologically without disrupting homeostatic Wnt regulation in normal tissues.

## 5. Conclusion

This study establishes a conceptual and analytical bridge between inherited genetic variation, lactylation‐associated epigenetic regulation, and single‐cell TME organization in CRC. By identifying AXIN1 as a germline variant–supported, myeloid‐enriched candidate linked to inflammatory and stromal remodeling programs, we provide a focused entry point for dissecting how metabolic‐epigenetic circuits may be genetically wired to influence CRC susceptibility and progression. As the field of onco‐metabolism continues to evolve, integrating germline genetics, single‐cell multiomics, and spatially resolved functional assays will be pivotal for translating lactylation biology into precision prevention and therapy strategies for CRC.

**Nomenclature**
CRC: colorectal cancer
TME: tumor microenvironment
GWAS: Mendelian randomization
SNP: single nucleotide polymorphism
eQTL: expression quantitative trait locus
GWAS: genome‐wide association study
scRNA‐seq: single‐cell RNA sequencing
UMI: unique molecular identifier
UMAP: uniform manifold approximation and projection
PCA: principal component analysis
IVW: inverse‐variance weighted
OR: odds ratio
CI: confidence interval
GO: Gene Ontology
BP: biological process
KEGG: Kyoto Encyclopedia of Genes and Genomes
FDR: false discovery rate
cDC: conventional dendritic cell
NBS1: nibrin; SPP1: secreted phosphoprotein 1
MIF: macrophage migration inhibitory factor
APP: amyloid beta precursor protein
FN1: fibronectin 1
ITGA5: integrin subunit alpha 5
ITGB1: integrin subunit beta 1
NAMPT: nicotinamide phosphoribosyltransferase
PPIA: peptidylprolyl isomerase A
BSG: basigin
AMPK: AMP‐activated protein kinase
mTOR: mechanistic target of rapamycin
Wnt: wingless‐type MMTV integration site family
*β*‐catenin: beta‐catenin
YAP: Yes‐associated protein
TAZ: transcriptional coactivator with PDZ‐binding motif
IHC: immunohistochemistry

## Author Contributions

Biao Sheng: conceptualization, methodology, software, formal analysis, data curation, writing—original draft, visualization. Jianwei Wang: conceptualization, validation, resources, writing—review and editing, supervision, project administration.

## Funding

No funding was received for this manuscript.

## Disclosure

Both authors have read and approved the final version of the manuscript.

## Ethics Statement

All data used in this study were obtained from publicly available databases, including the IEU OpenGWAS database and the GEO database. The original studies had obtained ethical approval and informed consent from the corresponding institutions and participants. This study did not involve newly collected clinical samples or identifiable personal information; therefore, additional ethical approval was not required.

## Conflicts of Interest

The authors declare no conflicts of interest.

## Data Availability

The data that support the findings of this study are available from the corresponding author upon reasonable request.
